# An Essay on the Summer and Autumnal Remittent Fevers of Mississippi

**Published:** 1840

**Authors:** John W. Monette

**Affiliations:** Washington, Mississippi


### WESTERN JOURNAL

OF

### MEDICINE AND SURGERY.

FEBRUARY, 1840.

### Art. 1.—*An Essay on the Summer and Autumnal Remittent Fevers of Mississippi.* By John W. Monette, M. D., of Washington, Mississippi.

The following remarks are applicable especially to the endemical fevers of the old Natchez district, embracing the country within forty or fifty miles of Natchez, on the east side of the Mississippi. We apply the term "Remittent" merely as a general characteristic appellation, without any other signification, as nearly every case of our summer and autumnal fevers have exacerbations more or less regular, whatever other type they may assume.

After a few pathological remarks, I shall proceed to speak generally of the *nature, symptoms, pathology and treatment* of the different varieties of the two great classes of fevers in the south, viz.: *Fevers of open excitement, and congestive fevers.*

### PATHOLOGICAL REMARKS.

According to Fordyce, "Fever is a disease of the whole system; it affects the head, trunk of the body, and the extremities; it affects the circulation, the absorption, and the nervous system; it affects the skin, muscular fibres, and the membranes; it affects the body, and it affects likewise the mind." Certain influences may so modify the acting cause as to produce cases of disease apparently distinct in character; and of these modifying influences, which cause these fevers to assume different types, the most common are difference of age, sex, temperament, climate, season, degree of predisposition pre-induced by exposure, and fatigue; besides other circumstances too tedious to rehearse. These often cause fever to assume the various characteristic forms designated by appellations, which seem to indicate distinct and opposite diseases. At least such is the case with those forms of fever which are most prevalent in the State of Mississippi, (and probably in other southern portions of the United States,) during the summer and autumnal months.

In a pathological point of view there may be propriety in the division of fevers into intermittent, remittent and continued, because there appears to be something peculiar, especially in intermittents; some peculiar property, or identifying principle, which makes them a class *sui generis;* but in a therapeutic point of light, the distinction is inadmissible. Autumnal fevers so often assume the remittent, intermittent, or continued forms, and so often pass alternately into each other, that the common diagnostic appellation is insufficient to satisfy therapeutic accuracy. A fever may be remittent or continued, and yet possess the same important features; the same visceral congestion; the same broken reaction; the same local inflammation, or the same general excitement: or different cases grouped under the same class may each present very important diverse indications of cure.

In southern latitudes the influence of a tropical sun appears to cause all fevers to assume the remittent form, whatever other peculiarities may attend them. The same influence, also, seems to accelerate the morbid action, and hasten them on to fatal terminations, or to the most dangerous extremes, if not speedily arrested. Hence in this latitude almost every case of summer and autumnal fever excites serious apprehensions, and not unfrequently sets at defiance professional skill, and the powers of medicine.

Fevers of the remittent class constitute, under numerous varieties, a large majority of all the diseases to which the inhabitants of the south are liable, during nine months in the year. Even in the midst of winter, such is the mildness of the climate, many cases assume the characteristic symptoms of autumnal diseases. Those cases which occur in the autumnal months, as a general rule, are more malignant in their character, and of course more difficult to manage to a salutary issue. Fevers of inflammatory character are more common in January and February.

The predisposition to fever is stronger as a general rule in natives and those who have been some years resident in the country, than in those who are recently from more northern latitudes; provided the latter take the necessary precautions to avoid all improper exposure and imprudent exciting causes. It appears that the tone and tension of fibre existing in a northern constitution, resists the febrile influences, especially those of a debilitating or relaxing nature; while the natives with their systems already more relaxed and enervated by the climate, are obliged to be more observant of those rules which they have found necessary for their health and comfort. Hence, also, strangers during their first summer, if prudent, are attacked with only a single fever of general excitement, which is easily subdued by depletion and the usual antiphlogistic regimen; while if attacked after their second or third summer, depletion will not be so requisite: and at a still later period after their emigration from the north they will be more liable, when attacked, to fevers of a *congestive* character, as they assimilate in constitution to the native.

In severe attacks of our autumnal fevers, the prominent symptoms often simulate those characteristic of specific or particular diseases; and the inexperienced practitioner is not unfrequently deceived and led into erroneous treatment. The most frequent of these appearances are those of cholera morbus, dysentery, bilious diarrhœa, menorrhagia, enteritis, cephalitis, and others which supervene in the progress of the disease. Even tetanus when occuring in summer and autumn, may not unfrequently be the result of that disordered state of the system, which generally shows itself in the developement of fever: and in such case the treatment adapted to that pathological state of fever in which tetanus appears, will be applicable to that manifestation of the morbid influence.

In an irritable constitution, the irritation may fall upon the liver, and profuse bilious discharges will be the consequence; or the brain will suffer in an equal degree: in a relaxed and leucophlegmatic temperament the irritation with relaxation may settle upon the alimentary canal, and profuse watery discharges will ensue; and unless arrested will induce visceral congestion and collapse: in a plethoric habit and in females especially, when a remora has formed in any viscus or organ with a hæmorrhagic diathesis, the disease will be first indicated by hæmorrhage, as in menorrhagia, hæmorrhoids, &c. In another constitution, with firm tone of fibre, the circulation becomes excited, and reaction with partial inflammation takes place in some membrane or tissue, such as in enteritis or phrenitis: the irritation may be communicated to the spinal cord and nervous system, and symptoms of tetanus may succeed. In such cases it is the duty of the physician in attendance to ascertain with discrimination how he may best and most speedily relieve the general as well as the local effects of the morbid influence. In these views I am fully sustained by some of the best writers upon the diseases of tropical climates; than whom none is more to be approved, than Dr. James Johnson; from whom I will make a few extracts. I will however premise that he is too much filled with the impression that the liver is the only organ which suffers, and is the only one deserving our attention, almost to the exclusion of all the other great sub-systems.

Dr. Johnson observes, "in every case of dysentery, that has ever come within the range of my observation, two functions were invariably disordered from the very onset, and soon drew other derangements into their train. These were the functions of the skin, and of the liver; or perspiration and biliary secretions."—(*Trop. Clim.* p. 331.) Again—"The equilibrium of the circulation and excitability becomes disturbed. In consequence of the torpor in the extreme vessels on the surface, the volume of blood is directed to the interior; and the balance is further broken by the check which the portal current meets in the liver, from a corresponding torpor in the extreme or secreting vessels of that organ: the effect of which is, that the plethora in the cœliac and mesenteric circles is now greatly augmented and febrile symptoms commence."—*Ib.* 332.

The form of the febrile action which is developed will vary according to the constitution, and habit of the patient, or the predisposition existing in certain organs; for, as Dr. Johnson continues "the same causes, that, applied to one person, produce bilious fever, will in a second give rise to hepatitis; in a third to *mort de chien;* and in a fourth to dysentery; according to the organ that happens to be most predisposed to disease. * * * In short, the *same general causes* produce *bilious fever, hepatitis and dysentery.* They are branches from the same stem; the *organs principally affected* occasioning the variety of aspect: * * * and the principle which is to govern us, is, the restoration of the healthy perspiration and the biliary secretion, with the equilibrium of the circulation and excitability." In other words, "they are all cured on the same principle, and with some slight variety arising from local circumstances, by the same remedies: a strong proof of the connexion I have traced."—*Ibid* p. 345. We readily admit that the liver is generally the first great organ to sympathise with the function of the skin; but other organs and tissues likewise take on this sympathetic action; sometimes the bowels—sometimes the peritoneal covering of the abdomen; sometimes the nervous membrane of the stomach and bowels; even the uterus, in certain conditions sympathises more intimately than the liver. How this mysterious sympathy of function and condition between the skin and internal organs, is effected, is not within our power to explain; nor is it necessary; for the fact as it exists is all that is necessary, to comprehend the proper method of cure.

As Dr. Johnson has properly observed, whatever organ is first implicated, if not relieved, soon draws into its train others successively, until the whole system is as deeply implicated in the disordered action as the first; and the general circulation, the secernent functions, and the nervous system are equally diseased. The *onus morbi* finally falls upon the weakest member, and thus the character of the general disease often changes its type in its progress: the nervous system may suffer in the advanced stages of the disease, although the sanguiferous system was most implicated in the first stage. The constitutional predisposition may cause cases to differ, although the locality and the exciting causes are the same.

It is not my object to discuss the morbific properties of miasm under its different varieties: but I doubt not, the most frequent sources of tropical diseases, are atmospheric influences operating through the medium of the skin. These influences are the changes of temperature and changes of humidity; or both irregularly succeeding or alternating each other. Much misapprehension and obscurity rest upon the doctrine of miasms; which are far less efficient as predisposing and exciting causes of fever, than solar influence; operating directly or indirectly upon the body. The sympathy of function and circulation existing between the surface of the body, and the various internal organs and tissues, gives rise to the various trains of morbid action which constitute our autumnal diseases. For the intimate connexion between the surface and internal organs and tissues we refer the reader to Dr. Johnson's works, upon "Tropical Climates" and "on the Liver." As he has treated very fully on this subject, we shall not detain the reader with a reiteration of those ideas which are so fully elucidated in the works just named. In the various forms of remitting fevers, the identity of the disease consists in the following series of symptoms variously combined; and these produce according to their diversity of combination in different cases, the apparent diversity of disease, viz: disordered cutaneous and hepatic secretion; venous engorgement of the viscera, with diminished external heat and circulation; or arterial excitement with increased heat of the surface; broken reaction, or irregular determination of blood, with arterial excitement; irregular distribution of nervous influence, with pain or insensibility; diminished secretion from over excitement, or from congestion, attended with organic torpor; increased or diminished nervous irritability, with general torpor or spasmodic contractions of the muscles of locomotion. These are the general leading symptoms which constitute by far the majority of cases of summer and autumnal diseases in this latitude. Different cases consist of different groups of organs drawn into the disordered action; and each affected in its own peculiar manner, and inducing other sympathetic action; hence too often the great apparent diversity of diseases, which are in fact only so many modes in which disordered action makes itself cognizable to our senses. To explain satisfactorily the *rationale* or modus operandi of all the consecutive actions is, and will remain beyond the ken of human research. Nervous influence by nervous connection will explain much but not all: there are links of morbid action, and organic derangement which we cannot explain by any theory now known. Yet the fact that these sympathetic and functional links have an existence, frequently serves to direct our attention to the most salutary therapeutic course, and even to remote organs.

It has been contended by some that these febrile affections, but more especially yellow fever, depend upon an inflamed condition of the investing membranes of the spinal cord, the ganglious and nervous filaments. But whether this be the case or not, whether the investing membranes be inflamed or congested; whether functional action be disordered through the nervous filaments, by conveying to the parts upon which they are distributed, a morbid nervous influence from the brain; or whether the brain as a *sensorium commune*, received disordered impressions through the nervous distribution, we deem but idle hypothesis and speculation; for whatever be the condition of these nervous filaments, it is but a link in the chain of disordered action; and in a therapeutic point of view it does not in the least facilitate the cure or modify the treatment. Our attention must be given to the great chain of morbid action as developed in the general system. In those sudden and violent cases, properly *congestive*, there is a collapse of the circulation similar to that in cholera maligna, but *not* with the same exhaustion of the circulating fluids. In congestive fever the *collapse is in the nervous and cerebral systems; the cerebral and nervous influence is prostrated as a primary effect;* and this may be roused through the medium of other tissues.

In most of our rapid and alarming cases of summer and autumnal fevers the stage of *oppression* is one of great importance; for while this stage is in progress, and it continues from a few hours to three days, the peculiar type and grade of the subsequent fever is established. If the system be plethoric of vital and other fluids, but of feeble powers of reaction, and weak nervous energy, the brain and nervous system are at once, or gradually overwhelmed, and reaction in the circulation, and cerebral energy are both imperfect. This is evinced by a greater or less degree of languor, listlessness, apathy, with relaxed skin and feeble circulation. The viscera of the three great cavities, from imperfect powers of reaction, become surcharged with blood, which scarcely circulates at all; while the general circulation is feeble and exhausted of a large portion of the general circulating mass. The skin becomes more relaxed, cool and marble-like to the sight and touch; the brain becomes more languid, the great hepatic secretions are suppressed, and all other important secretions are equally arrested. A few hours often suffice to tell the melancholy tale. This is a case of rapid and fatal *congestive fever.* Those cases which are less fatal are attended with more suffering, and to those unexperienced in them, more alarming symptoms; which are only evidences of stronger constitutional powers of reaction. Those symptoms which generally give a favourable indication of the powers of the system, are extreme pain in the spine, and extremities; extreme irritation of the stomach and even of the larger intestines; a sudden discharge; of yellow or green bile; and in females, of a morbid menstrual discharge; sometimes the irritability is confined to the liver and results in copious secretion and discharge of yellow bile; which is always a favourable occurrence, if properly restrained and controlled; otherwise it may prove a means of rapid exhaustion. This is the great flood-gate by which the system is often relieved in these congestive cases. When the reaction is free, or where there is constitutional stamina for reaction, and aromatic and other active stimulants have been freely and perseveringly used, it is not uncommon for a degree of inflammation to succeed in the mucous membrane of the stomach and alimentary canal; when the opposite mode of treatment must be adopted. In the same manner local inflammations may supervene in other organs, in the progress of the change towards a salutary termination. Yet there is no case within my recollection where congestive fever has terminated in a stage of open excitement; or where the moderate reaction, which sometimes occurs, would not be most injuriously arrested by even moderate bloodletting.

In the successful treatment of these types of fever, it is a matter of great importance to observe carefully the peculiar modes of developement, and the grades and order of sequence in the action. After the circulation is partially restored, the great object is to equalize and moderate local irritations; to control or moderate sympathetic sensations; to restore, correct, and moderate the secretions, and to evacuate morbid secretions from the alimentary canal; and thus obviate other sources of irritation.

To accomplish these ends, such remedies and medicines should be used, as are known by experience to operate in a favorable manner upon the organs or tissues most implicated in the morbid derangement, with as little disadvantage as possible, to others which are not disordered in the same degree, or which are not in the same pathological condition. In most cases of summer fever, whether it be an original case of congestive fever. or some variety of fever with open excitement, the nervous system suffers much in the progress of the disease; and the use of anodynes, and narcotics must not be overlooked; as is too frequently the case from prejudiced views. We shall further speak of these fevers common in Mississippi under the heads of *fevers of open excitement, and congestive fever.* This division if not more correct and philosophical, is at least better adapted for practical purposes, and the remarks which I propose to offer, than is the old and received division into intermittent, remittent and continued fevers. In these remarks, I desire it to be clearly understood that I do not confound these fevers with the *y llow fever* which sometimes is epidemic in our cities; for having seen much of both, I have no hesitation in declaring that I believe the latter a malignant disease *sui generis;* a disease which in its worst forms upon unacclimated persons is as much beyond the control of medicine now as it was in the days of Dr. Rush.*

As it has been frequently asserted by physicians who have had partial opportunities of observation in the south-west, that typhus fever is not known in this latitude, I will make the passing remark that I have often seen in the latter part of autumn and in the early part of the winter season, cases of *typhus mitior* as genuine as it occurs in more northern latitudes, but rather shorter in duration, and more violent in its access and progress.

We will first speak of fevers of excitement, with some practical remarks on the most judicious mode of treatment; after which we will speak more fully on congestive fever.

### FEVERS OF EXCITEMENT.

In this class I comprise all those cases of common summer and autumnal fever, in which increased arterial action or excitement, and increased heat of the surface are the most prominent symptoms, during the *early stage* if not throughout the whole progress. Yet cases do not always preserve the same character through all their stages: in a case where the excitement appears general and equally developed, some organ, such as the liver or spleen, may labour under a languid circulation, not unlike congestion; while other organs. may yield to the continued excitement and inflammation, or great irritation may supervene. These consecutive symptoms may supervene in the mucous membrane of the alimentary canal,

* The epidemic so easily manged by Dr. Rush was "*Bilious* Yellow Fever"—a milder grade of that disease.

in the peritoneal coat of the abdomen, in the membranes of the brain, &c.

The general symptoms of this class of fevers, especially in the early stages are as follow, viz: The pulse is frequent, full and soft, or frequent, full and tense; the skin is hot, dry and florid, or hot dry and pale; and sometimes hot and moist, where abdominal congestion prevails; the tongue is pale and moist, or florid with numerous clean red points in the midst of a whitish or drab fur; sometimes the tongue is covered with a brown, or russet fur, but moist; at other times it is covered with a brown fur and dry or chapped; at other times the tongue is clean, red and glabrous, or dry, red and chapped; the secretions of bile, urine, &c., are vitiated or entirely suppressed, especially the former; the bowels are at first constipated, but often relaxed; the alvine discharges at first a dark yellow, or muddy yellow; upon the use of cathartics changing to a yellow serous fluid, or to pure yellow bile; there is more or less pain in the epigastrium or head; great inquietude, with mental confusion or delirium, and sometimes great irritability of the stomach. Where the pain in the head, spine and extremities is severe, with copious discharges of yellow bile, and with great nervous irritability, the case is properly one of *bilious fever.*

The genuine *bilious fever* of hot climates is simply a remittent fever, more or less violent, engendered upon an enfeebled constitution and irritable condition of the whole system, nervous and vascular. In these cases, especially where the pulse is quick and irritable, the degree of *irritation* in the nervous and vascular systems is proportionate to the irritable excitement; and this excitement must be reduced by calming the nervous system, and not by copious depletion. Moderate depletion with a judicious administration of opiates and narcotics will succeed best. When the great emunctory of the system, the liver, partakes largely of the general irritation, it is thrown into excessive functional action; which is indicated by the profuse discharge of yellow bile; whereby the system is depleted, and still more exhausted, while the general irritability increases with the debility thus induced.

It is not uncommon for all cases of summer fever to be termed bilious fever; and a still more erroneous therapeutic course of treatment is the result. Instead of general portal torpor in *bilious fever*, as exists in many cases which are also termed bilious, we have a general morbid *irritable excitement*, and a peculiar train of sympathetic affections dependant upon an excited and irritable state of the whole portal circle, and of the brain. The brain, whether primarily or consecutively affected, is strongly implicated; and the intense pain in the head, in bilious fever, is no less a characteristic symptom than the profuse discharges of bile.

The characteristic symptoms of *bilious fever* are a quick, soft, and irritable pulse; great restlessness and anxiety; intense *pain in the head* with intolerance of light and sound; *profuse vomiting of thin yellow bile*, and often copious discharges of the same through the intestines; the skin is hot but without the dry mordant sensation produced by some other fevers; often the skin is bedewed with sweat and tinged of a dusky yellow; the stomach becomes very irritable, and other symptoms present in the progress of the disease, and vary with the modes of treatment and other contingent circumstances. The worst cases are in children from seven to sixteen years of age; and in those of lymphatic and nervous temperaments, whose system has been debilitated by previous indisposition, or visceral enlargements, or by poverty in diet and exposure to inclement weather. In such the countenance is indicative of extreme anxiety, unless the cerebral irritation has been superseded by stupor; the skin pale and hot, and bathed in sweat; the abdomen is somewhat distended and inelastic: purgatives at first seem slow to act; but if active cathartics are administered they excite extreme mucous irritation in the bowels; copious serous and bilious discharges are thrown forth, and unless allayed by opiates and other anodyne remedies, bloody mucus succeeds, and exhaustion and death supervene. This class of fevers is more common in the lowlands along the Mississippi.

Under all their different forms and grades, these fevers appear to observe regular remissions corresponding to the paroxysms of quotidian and tertian intermittents; and the remissions are more complete where there is less of abdominal congestion or irritation. Thus the remissions in cases of true *bilious fever* are more complete than in other varieties: not unfrequently the patient that in the morning was free from fever and strongly anticipating health, in a few hours afterwards is despondent in all the sufferings of another aggravated paroxysm. Bilious remittents appear to observe regular quotidian or double tertian type; each alternate day being more or less severe: the whole duration of a regular attack well treated, does not exceed from three to seven days. However in some cases both during the hot months as well as the cool or winter months, these cases may be protracted to ten or fifteen days. The duration is in proportion to the intestinal irritation induced in each case by the disease and remedies.

### TREATMENT.

In the treatment of these fevers, it would be absurd to lay down any uniform plan adapted to all cases; the judgment and observation of a discriminating mind are at all times requisite to adapt the remedies and treatment to the varied conditions of the system, as they alternately present themselves, and imperiously require corresponding changes in the course of treatment. The enlightened physician will take a comprehensive and pervading view of the whole system, as formed of many sub-systems, which in health must harmonize in all their actions: when he sees discordant actions in any one or more of these sub-systems, his attention must be attracted to that which is most likely to exert the most periodical influence upon the whole. To treat a case properly he must know not only the extent, but the peculiar nature of the derangement in each organ or function in all the stages and periods of the disease ; for in this latitude the changes in severe cases are often rapid, and must be observed at the earliest moment. None but an ignorant empiric would attempt to treat these diseases under a certain name, and by a prescribed routine of practice. One important distinction to be made is between *active* excitement and *irritable* excitement, whether in the circulation, or in the tissues ; for in persons of delicate constitutions, and in females, the latter generally predominates ; and is to be allayed by any other means than depletion and the active antiphlogistic regimen. A close discriminating eye must be kept upon the relation between the healthy and the morbid physiology of each and every sub-system in the united whole. Without this close observation, followed by a judicious, decisive and prompt treatment, the physician will often have the mortification to find his patient beyond the reach of medicine, before he has even apprehended danger. The progress of violent attacks of fever in this region, is so rapid, that there is but little time to be lost by indecisive treatment, or in trusting to the powers of nature.

The treatment pursued in the East and West Indies, with appropriate modifications according to circumstances of constitution, of habit, and of nativity, will answer in Mississippi as a general rule.

The excitement should be reduced as promptly as possible.

1st. *By bloodletting.*—If the patient be young, plethoric and athletic, or from a northern climate, with a full, bounding, strong pulse, bloodletting by the lancet to the amount of fifteen or twenty ounces in a sitting posture, from a large orifice, will produce the most immediate effect upon the arterial excitement. The object should be to produce as great reduction of the excitement as possible with as little loss of blood as practicable; hence twelve or fifteen ounces of blood rapidly drawn while the patient is in a sitting posture, will in most cases speedily induce a tendency to syncope, with a sudden reduction of the pulse. During the flow of blood the approach of this state is to be observed closely, and the patient should be placed immediately in a recumbent posture, and the arm tied up. If the system is very plethoric, and the constitution vigorous, in half an hour or an hour the pulse will regain its former strength and action; when the orifice is to be again opened and ten or twelve ounces more drawn as before. The pulse will yield sooner than at first, and its reduction will be more permanent and effectual; for it is seldom if ever necessary to have recourse to the abstraction of blood oftener than twice. If the pulse becomes active and quick in its pulsations, after the second bleeding, we may be assured we have already taken more blood than was required; and thereby have induced the *irritable* excitement instead of the open excitement. Hence the great advantage of taking blood rapidly, and in an erect or sitting posture in the first instance; so as to produce a strong impression with the loss of as little blood as possible. The slightest irritation in the pulse after bloodletting should be allayed by a full anodyne of fifteen or twenty drops of laudanum, or 1-6 to 1-4 grain morphia, either alone or combined with ten or fifteen drops of tinct. hyosciam, nig: So soon as the patient recovers from the depletion, and the anodyne has been administered one hour, an active cathartic should be given in moderate and repeated doses. If the alvine dejections indicate a defective secretion of bile, with serous or mucous discharges and coated tongue, calomel and jalap will be preferable to most others: if the alvine dejections are frequent, and consist principally of thin yellow bile, the mild saline purgatives are best. Of these, in such cases the common dose of infusion of senna fol. manna, and sul. magnesia, divided into repeated broken doses, is superior to most others. The operation of course should be promoted by a free use of diluents. This purgative is infinitely superior to simple saline or resinous purgatives in simple bilious fever.

If the excitement be of the irritable kind, attended with a quick pulse, moderately full but not tense, the excitement should be allayed by anodynes and narcotics, to the entire exclusion of bloodletting, which would only aggravate the irritable excitement. So soon as this is in some degree accomplished, the use of laxatives or mild purgatives should be commenced. Drastic purgatives will tend to excite greater irritation in the alimentary canal and aggravate the general irritable excitement. In this irritable excitement, if profuse *bilious* discharges are such as to debilitate, drastic purgatives should be avoided; and opiates combined with mild saline laxatives should be administered; and thus repeated in moderate doses every hour for five or six hours, or until six or seven drachms of sulph. mag. and fifty or sixty drops of laudanum, have been taken, we obtain a more salutary evacuation of the bowels, while the irritability is allayed.

On the other hand if this irritable condition of the nervous and arterial systems is attended with a *torpid* condition of the bowels, and with a suppression of biliary secretion, our purgative should consist principally of calomel combined with some powerful aromatic, such as capsicum, zingiber, or oil of black pepper. This rouses up the circulation and sensibilities of the stomach and bowels, while the calomel exerts its influence upon the functions of the liver. When it is necessary to administer calomel for suppressed biliary secretion, it should be administered in doses of ten or fifteen grains every two hours until the secretion of *green bile* is excited; when the further use of calomel should cease, and be succeeded by a gentle saline laxative or oil. In this administration of calomel, if the tongue be moist, and pale, or white, the addition of four or five grains of capsicum will facilitate its salutary effects upon the torpid liver.

I have thus dwelt more upon the use of stimulants and of narcotics or anodynes under this head, than upon bloodletting itself, because by their means the very object is effected in a great majority of cases, which would in vain be expected from bloodletting which is so often and so injudiciously used, in the early stages of our autumnal fevers.

There are other cases where the pulse is partially oppressed, and where the blood is thick and viscid, in which bloodletting, judiciously used, will prove an important remedial agent. In what cases and under what circumstances this should be resorted to, it is not my object to point out: elementary works on that subject give all the necessary rules for discrimination. My object is principally to guard inexperienced practitioners from being deceived in the necessity of *frequent* bloodletting in many of our autumnal fevers; when *irritable excitement* is often mistaken for open sthenic excitement. When such mistake is made, the worst consequences may be apprehended; for every drop of blood drawn in such cases only adds to the irritable excitement already established. It is in vain, in those cases, to expect to reduce the action of the pulse by repeated bloodletting; for the mistaken irritable excitement becomes more aggravated after each bloodletting. Opiates are the only resource in such cases, if we except that important auxiliary, bathing the head in cold water. In many of these cases it will be essentially necessary to the welfare of the patient, to administer the anodyne in a small quantity of claret and water, weak brandy toddy, or some other stimulus, to sustain a proper circulation and tone in the stomach.

2. Emetics are often indispensable remedies in autumnal diseases: yet they should be used with great caution. Gastric and gastro-enteric irritations are so frequently consecutive symptoms in these fevers, that it is not without reason that many authors in the treatment of tropical diseases, almost discard them entirely. It is, however, not necessary to abandon a good remedy because some have used it improperly, and consequently produced injurious effects. A valuable remedy requires only more judgment in its administration. As gastro-enteric irritation or inflammation is of so frequent occurrence in the advanced stages of our autumnal fevers, emetics, as a general rule, should be used only in the first stage, while there is no structural disease in the tissues of the alimentary canal.

Where there is no *permanent* gastric irritation, or visceral inflammation, and where there is evidence of morbid secretions in the stomach, an emetic is certainly the first remedy that should be employed. In such case, even if the excitement be open and strong, an emetic will be the most suitable remedy with which to begin the treatment; as the irritating matters being removed, the excitement may be reduced without unnecessary depletion. If the emetic is given during the first paroxysm, before the disease becomes confirmed in its character, the advantages derived from its operation will be far more important than when given in the second or third paroxysm: it thus not only evacutes the morbid secretions before they have produced *permanent* irritation, but it breaks up the morbid chain of associations, determines to the surface and equalizes the circulation. However, it is seldom necessary or proper to repeat the emetic in less than forty-eight hours, and generally not then.

When emetics are to be used, nothing is preferable to ipecacuanha or tartrite of antimony; the former in a single full dose, or the latter in small and repeated portions. After their operation is over, it will always be advisable to administer a mild anodyne, of fifteen drops of laudanum, or one-fourth of a grain or less of morphia. This will have a salutary effect in preventing the distressing irritation which is so apt to supervene in these fevers. Sometimes the stomach is irritable early in the disease, with constant retching and efforts to vomit, without discharging freely, although morbid secretions may exist; in such cases, if the tongue *be moist and whitish*, or covered with mucous, an emetic of ipecacuanha will often produce an immediate relief to the *irregular* action of the stomach, and be succeeded in less than half an hour by free vomiting. This I have witnessed so often, that I have, for several years, used ipecac. either in full doses, or in minute portions of half a grain every half hour, to allay the most obstinate gastric irritation. If the irritation has been of short duration, the emetic in full dose is preferable; but if it be of some duration, with red tongue, the minute doses, with a small portion of morphia or laudanum, are preferable ; and very often the same added to a weak solution of epsom salts, is still better. The following solution I have used with great advantage in those cases of febrile diseases in which the tongue becomes red, clean and dry ; or red and moist, indicative of different grades of sub-acute inflammation of the mucous membrane of the stomach, viz:

℞. Sul. mag. ʒij. or ʒiij.

Sulph. morphiæ gr. ij.

Ipecac. pulv. gr. vj.

Aqua pluvial: f℥vj. misce.

Dose, one table spoonful every hour or every two hours p. r. n. Sometimes it may be advisable to diminish the quantity of morphia, and give the dose every half hour, especially where the tongue is moist and coated white. If it be desirable to produce a laxative effect, the sulphate of magnesia may be increased.

Sometimes, in protracted cases of fever inclining to the congestive type, the stomach becomes torpid and charged with mucus and other morbid secretions, which not only act as a new source of irritation, but also prevent the salutary operation of medicines. In such cases the tongue is generally moist and covered with a thick brown fur, indicative of torpor in the stomach, although slight irritation may exist in the lower intestines. In such cases the operation of a brisk emetic is essentially necessary to discharge the morbid secretions, and to excite action through the large viscera of the abdomen ; and yet, were a full dose of ipecacuanha administered without any preparatory steps, we might produce a sudden collapse of the weakened powers of life ; or the emetic, failing to act speedily upon the stomach, might pass off by the bowels, and thus produce exhaustion. To guard against either of these effects, I have prepared the stomach and general system by the following means, viz. by administering a wine-glassful of hot strong toddy, rendered pungent by the addition of capsicum or ginger, about fifteen or twenty minutes before the administration of the emetic, which should be given in one full dose, so as to produce its emetic effects suddenly without in any degree prostrating the vital powers of the system. I have pursued this plan with the most remarkable benefit in cases where the treatment had been temporising and the disease protracted; and yet, from the torpid condition of the stomach, and languid circulation, I am convinced that, without these precautions, I should have produced exhaustion or collapse without vomiting.

Where there is relaxation in the skin, langour in the circulation, accompanied with profuse biliary discharges, the free use of emetic medicines is improper, whether administered in full or broken doses. As Dr. Sanders, (on Liver, p. 176,) "In all cases where bile is secreted in too large a quantity, the use of emetics is improper: indeed, the action of nausea and vomiting increases its secretion." Dr. Johnson confirms the same by his testimony; he says, "Every one knows the effects of emetics and nauseating medicines upon the skin and perspiration; the same effects are produced upon the biliary secretion." (Trop. Clim. vol. 1, p. 275.)

Where the open excitement continues in the circulation, after proper depletion by bloodletting and purgatives, it will be advisable to administer the tartrite of antimony in broken doses, to keep up nausea and a determination to the skin, provided the biliary secretion be not already too profuse. If this excitement continue, and the biliary secretion be deficient, the following antimonial solution will be excellent to excite secretion in the liver, at the same time that it reduces the excitement and determines to the skin.

℞. Tart. antimonii gr. 5.

Aquæ pluvial. f℥iv.

Tinc. Opii fℨiv. Mix.

Dose, fℨij., to iij., every two hours, or oftener in urgent cases, until nausea is induced, or until the bowels are moved freely. If the excitement be of the irritable kind, this of course is not applicable, unless the quantity of laudanum be increased.

3. *Cathartics.*—In treating these fevers, I have always found it much preferable to administer cathartics in divided doses than in one single portion. By this mode of administration we avoid producing that irritation in the alimentary canal which would result from the same quantity given at a single dose; and at the same time we obtain the operation upon the bowels more effectively than we should do by one large undivided dose. After the operation of the emetic I have found ten grains calomel and ten grains jalap, given and repeated in two hours, one of the best purgatives in ordinary cases. Sometimes I omit the emetic, and add half a grain of tartrite of antimony to each of the above doses. This combination is one of the best purgatives in ordinary cases, where there is no profuse secretion of bile, nor an entire suppression of that secretion. The following combination also is excellent, where there is more nervous irritation in the general system, and deficient biliary secretions, viz:

℞. Submur. hydrarg. ℥jj.

Pulvis Antimonialis ℨss.

Camphor. pulv. grs. iij.

Opii. pulv. gr. ij. Mix.

Divide into three equal doses—one to be taken every three hours.

Where the biliary secretion is excessive, the best purgative, after a full opiate, is the infusion of senna, manna and salts, taken in divided doses, every hour, until free purgation succeeds. This dose operates more effectually in removing the bile than any other, while it produces no effect upon its secretion, unless it be to restrain it. Indeed, in this variety of fever, the principal indication should be to *restrain the secretion*, while we evacuate the bile which is already secreted. The only way to restrain the secretion is to diminish the irritability of the liver and of the general system. This is to be effected by a liberal use of opiates and narcotics, judiciously administered. In these cases the administration of calomel is *exceedingly injurious*, as it increases the secretion, and thereby aggravates the disease.

When the excitement is violent, and the secretion of bile obstructed, we then must resort to the use of mercurial purgatives freely. One of the most valuable effects of calomel is its action upon the liver, in exciting biliary secretion; for which purpose it is more than equal to all other remedies we possess. If there be but little irritation of the bowels, and no serous alvine discharges, calomel, in doses of fifteen or twenty grains, combined with Dover's powder and camphor, may be administered every two or three hours, until *green gelatinous bile* is produced; but it should be discontinued so soon as this indication is perceived. I wish to enforce this point, as I have frequently seen the use of calomel persisted in, to the very great detriment of the patient, after this salutary indication of its action had presented. Whenever this *green, jelly-like bile* is produced, the calomel should be immediately discontinued, and some mild saline purgative administered to carry off this new acrid secretion. This being done, healthy yellow bile is sure to succeed. On the other hand, if the calomel be continued beyond that point, it begins to excite a state of irritation, not only to the mucous membrane of the bowels, but likewise to the general system; by which convalescence will be greatly retarded. This fact I wish to impress upon the reader, for I have seen many respectable practioners who were in the habit of administering calomel so long as the green discharges continued; without reflecting, and apparently without knowing, *that these green discharges were the real and salutary effects of the calomel.* In infants especially, by such perseverance in its administration, I have seen the mucous surface of the alimentary canal so irritated that it was barely possible to allay it by the most unintermitted exertions: when the use is continued too long, the brain and nervous system partake of the irritation, and the patient finally sinks under the combined influence of disease and medicine.

Equally injurious effects have I witnessed in that irritable disease malignant *bilious fever*, in persons of intemperate habits. One case in particular is in point. The patient was a man about forty years of age, who had been addicted to occasional intoxication, besides a common free use of ardent spirits; he was attacked with bilious fever; the prominent symptoms were profuse discharges of thin yellow bile; extreme pain in the head, constant vomiting, quick irritable pulse, great muscular debility and inquietude. Mercurial purgatives were used, and the symptoms increased; the calomel was discontinued and the discharges of bile and the general irritation were partially allayed with opiates. A consulting physician was called in, and insisting upon continuing the calomel in larger and more frequent doses, I reluctantly consented to give forty grains every two hours until the bilious discharges should cease. The first dose seemed to aggravate the symptoms; and after the second dose, the discharges of thin yellow bile by vomiting and catharsis was so extremely profuse, that speedy prostration seemed inevitable, unless it could be immediately checked. The calomel was immediately discontinued, and a large dose of laudanum administered by enema, as well as by mouth; this with stimulants and other means at length allayed the irritability of the liver and of the general system—and the patient finally recovered. In this case I do not hesitate to give it as my opinion that a few more doses of calomel would have proved fatal to this patient.

I do not presume to sit in judgment upon the errors of others, but I consider it my duty and my privilege to speak my sentiments freely upon the modern injudicious use of this valuable medicine. Of late calomel has been used and recommended so indiscriminately, and so injudiciously in all cases and in all grades of fevers, that it cannot be too severely reprobated by every judicious practitioner, who feels an interest for the honor of the profession or the welfare of his fellow men. It is to be regretted sincerely that such an extravagant use of it has been encouraged by writers and teachers whose high authority has given the practice a most mischievous currency.

From strict observation I am convinced that in all cases attended with *mucous irritation*, or inflammation in the alimentary canal, whether in bilious fever, or in dysentery, as an attendant symptom of autumnal and winter fevers, *calomel* is *decidedly injurious;* whether in children or in adults—but especially the former. The first impression of calomel is to excite the mucous membrane of the stomach and bowels especially of the duodenum, and induce an increased secretion of mucus to a greater or less degree; and when a patient is laboring under a disease of which this mucus irritation is the predominant symptom, it is certainly strange therapeutics, to give those very articles which produce and aggravate this condition. Any physician who will lay aside his preconceived notions, that calomel is adapted to every opposite condition of the system, and who will observe strictly the progressive effects of calomel in such cases, cannot fail to perceive the pernicious effects. Yet strange as it may seem under the use of calomel, it is too commonly the case, that every aggravated symptom, and each progressive step in the disease, are considered only so many evidences of the *necessity* of perseverance in the administration of this medicine, to arrest the intractable disease. Cases of this kind are fresh in my mind, where, in violent cases of autumnal fever attended with extreme irritation of the stomach and bowels, calomel has been given in form of pills to the extent of forty grains every two hours, notwithstanding the irritation excited by each dose was such as to cause the stomach to reject and throw up these heavy pills from one to twenty-four hours after they had been swallowed, enveloped in tough mucus, thrown out by the irritated coats of the stomach to protect itself from the poison. Calomel is often given likewise to children and infants in doses double as large in proportion, where the disease is intestinal fever, attended with extreme irritation throughout the alimentary canal. Children not one year old frequently are compelled to swallow from fifteen to twenty grains of calomel every two or three hours for several doses; and often I have seen the most distressing cerebral irritation superinduced by its use; yet when this irritation was produced, it was termed hydrocephalus acutus, for which calomel alone was considered an adequate remedy. Cases of infantile fever with intestinal irritation are the most frequent diseases to which children are subject in this climate; and in the advanced stages the particular symptoms are varied much by the treatment. A free use of calomel will be attended and followed by a train of symptoms depending entirely upon intestinal irritation inducing *cerebral* and general irritability. The most violent and rapid case of hydrocephalous symptoms which I have ever seen was produced by extreme cerebral irritation dependant upon intestinal irritation. The contracted pupil, jactitation, intolerance of light, frequent dejections of bloody serum, mucous and even blood in large quantities, and tenesmus, in this case, were relieved principally after many remedies had been unavailing, by cold affusion over the head, slowly and perseveringly used, together with anodynes by mouth and by enemata. The effect of the cold affusion was sudden, and astonished all who witnessed its effects; the infant had not rested five minutes in twenty-four hours and was supposed by all present to be only a few removes from its last struggles, when the affusion almost instantly calmed the cerebral and general irritability, the bowels even were allayed, and it slept soundly for more than an hour. Cases might be multiplied were it necessary. In August and September, 1838, a catarrhal bilious fever spread over a great portion of Adams County, beginning in Natchez, and was confined especially to children under five years. This was simply infantile *bilious fever*, with catarrhal symptoms superadded. The irritation primarily located in the bronchial tissues, upon the use of calomel speedily became transferred to the alimentary canal; and many fatal cases occurred, especially in the calomel practice.

There are other cases, where extreme irritation of the stomach proceeds from *duodenal inflammation*. It is upon the duodenum especially that calomel exerts its powerful influence in exciting the torpid liver to an active circulation, and secretion of bile: and when the *duodenum is inflamed calomel increases that inflammation*, and literally acts as a poison to its inflamed coats. The duodenum has a peculiar sensibility to calomel; and by this peculiar sensibility, does calomel exert its beneficial effects upon the liver. Hence when the duodenum is inflamed, nothing is more irritating to its coats than calomel. When the duodenum is inflamed, it produces the most deadly sickness and sense of prostration, with frequent efforts to vomit; tenderness over the region of the epigastrium; thirst, rejection of almost every thing taken, even many hours after it is swallowed; nothing seems to pass through the stomach, and the pylorus seems permanently constricted, so as to prevent the passage of all ingesta. If the duodenal inflammation be severe or protracted, the skin assumes a yellow tinge. This latter symptom indicates duodenal inflammation, whatever may be the general disease, whether *bilious fever, jaundice or yellow fever.*

So numerous are the cases, to the treatment of which calomel is not adapted, that I may be pardoned for dwelling more fully upon it, than might appear necessary with so trite a subject. It becomes every physician, who wishes to advance the interests and utility of medical science, to pause and reflect upon the injury which may be produced in his hands, by a presumptuous confidence in a remedy which cannot be adapted to every case; lest he be guilty of adding a destructive poison to the list of human woes, by converting a valuable remedy into a means of destruction.

It is too common for physicians who remove to the south to practice their profession, to bring with them, or soon afterwards imbibe the prejudice that calomel is the only remedy that is applicable to our summer diseases. To a certain extent this belief may be correct; but there are many exceptions. It is no uncommon occurance for such physicians to boast of the large amount of calomel given in the short period of twenty-four or forty-eight hours, in malignant fevers, and to express utter astonishment that it was possible for the patient to have died with such a quantity of calomel in them. It is no uncommon case for patients thus to have taken from one hundred and fifty to two hundred grains of calomel in twenty-four or thirty-six hours. I repeat that wherever there is duodenal inflammation calomel is not only improper but *injurious.*

When the duodenum is *not* inflamed, calomel administered perseveringly produces an irritated condition of the larger intestines, which is followed by an irritable condition of the whole nervous system: the bowels become charged with large quantities of green bilious matter and acrid mucous secretions, which alone are sufficient to produce the most distressing symptoms. Instead of mild laxatives and opiates to carry off the morbid or acrid secretions and allay the intestinal irritation, this green bilious matter is considered a strong indication for the further use of calomel.

There is another object for which calomel is used by those who practice in the south, viz: to produce ptyalism, or to produce its constitutional effects upon the system generally. Whatever be the disease, it is to be eradicated by mercury; whatever be the grade of fever the great object with them is to effect salivation, when the disease must of course yield. Yet who has not seen patients die of febrile diseases, while the system was charged with mercury? And how many have been compelled to linger out a miserable existence from its effects upon the constitution? The idea of curing a malignant fever by substituting the mercurial action, is fallacious and absurd. I fear not to hazard the assertion, that in our rapid autumnal fevers, it is impossible to establish the mercurial action, until the febrile action is first subdued by other means, or declines from having run its course. To establish the mercurial action after the febrile excitement is subdued, is only to protract the period of convalescence, by producing another morbid excitement, which the system must overcome.

Yet I repeat, calomel, when properly used, is one of the most valuable and efficient remedies in the materia medica. As has been appropriately said, it is the "Sampson of the materia medica, and like Sampson, it has slain its thousands," when its strength has been wrongly directed.

As a general rule, calomel is contra-indicated in all cases where the tongue is *clean and red;* whether it be smooth and dry, or smooth and moist; also, where the tongue is *dry and rough*, whether it be brown or red. In all cases these appearances of the tongue indicate different grades of mucous inflammation of the intestinal canal, and in all the use of calomel or quinine is decidedly pernicious to these surfaces.

4. *Cold Affusion.*--I place this remedy first in the list of febrifuges, for the treatment of our summer and autumnal fevers in their first stage, and even later. But especially is it applicable to such as are attended with open or irritable excitement and preternatural heat. In ardent fevers, the advantages of general cold affusion are too well known and admitted to require more than a passing remark; and it should be used under the circumstances and rules laid down by Dr. Currie and others. But in these remarks I wish to confine myself especially to the importance and utility of cold affusion *over the head*, as a powerful agent in the reduction of irritable excitement, as well as that which is more open. As has been observed before, many cases of our summer fevers seem to depend upon an irritable condition of the circulating system more than upon sthenic excitement. And this irritable condition doubtless proceeds from an *irritable condition of the brain*, although not often suspected. The *brain is* the principal seat of the irritation; and the circulation, and even the alimentary canal, takes on a consecutive irritation proportionate to the primary irritation of the brain; and when the irritation of the brain is allayed by cold affusion, these consecutive symptoms abate. It is not, in all cases of this kind, that the temperature of the head is greatly increased; for in these irritable cases of fever, especially in children, the temperature does not increase in proportion to the irritation, as is the case in inflammation. Yet, when the temperature of the head is reduced permanently, the general and local irritability ceases: and there is no mode of reducing the latter more certain and speedy than by reducing the former. Purgatives, antimonials and other febrifuges, may indirectly reduce the excitement; but not unfrequently they increase the irritability upon which that excitement depends.

In children, during the summer months, many cases of fever assume the general characteristic symptoms of the last stage of *cholera infantum*, and *hydrocephalus acutis*, in its most distressing form. Although the temperature of the head is but little increased in these cases, the irritability of the brain and nervous system is most distressing, and may often be speedily relieved by the affusion of cold water over the head. In one of the most rapid cases I ever saw, of a child six months old, the distressing irritability of the brain, attended with constant vomiting, with mucous and copious bloody alvine discharges, contracted pupil, constant gyration of the head, and even spasms, was in half an hour relieved, with all the attendant symptoms, by the affusion of *cool water over the head.* The irritation of the bowels subsided as soon as the affusion had been freely used to the head; although none of those symptoms had been alleviated by a persevering use of internal remedies for twenty-four hours previously.

Too much attention cannot be given to the condition of the brain in treating most of our summer fevers: and when the brain is found to be highly irritable, as a prime cause of fever, the simplest remedy in nature is the most effectual in reducing it; and that is cold water. With the aid of cold affusion to the head, we may bring the most alarming train of symptoms within the control of other remedies; where, without it, our remedies would be unavailing.

There are many obstacles to the use of general affusion in fevers, which are not applicable to partial affusion, or affusion over the head. I know of no insuperable objection to the latter, except when the system is charged with mercury; at such time cold water freely affused upon the head might be productive of the most distressing consequences.

The mode in which I use the cold affusion to the head, is, by pouring from the spout of a pitcher, in a very small stream, water of the ordinary temperature, between 64° and 80° Fahrenheit. The head is to be held over a basin and rubbed with a wet towel while the water is first affused. This causes the water to permeate the hair and come in contact with every part of the scalp. The affusion is continued in a very small stream passing the jet alternately over every part of the head for about five or ten minutes, when the head is to be wiped dry. The application of the water in this manner should be continued until the surface of the head is thoroughly cooled to the temperature of the water. If there has been great and obstinate heat in the head, the temperature will return as high as ever in half an hour; when the application of the water must be resumed without intermission, for ten or fifteen minutes, in a continual stream, as before. There are very few cases in which it will be requisite to repeat the application so thoroughly more than three or four times. After such reduction, the temperature will remain nearly at the natural standard, especially if succeeded by small opiates.

I consider it a material point to affuse the water *slowly*, so that it may gradually permeate the hair, and come in actual contact with every part of the scalp; and the stream or jet of water should be but little larger than what would pass through a common goose quill. The application of pounded ice, and cloths wet in refrigerated solutions, is not half so beneficial as the use of water at a temperature but a few degrees below summer heat; or about 70° or 80° Fahrenheit. When the temperature is at or below 60°, the shock is such that the patient is injured instead of being relieved; and the internal excitement of the brain is much better allayed by water at a higher temperature.

I must reiterate that, in *bilious fever*, the most prominent symptom is an irritable condition of the *brain and liver;* and that the primary seat of the disease is in the brain. To the irritable condition of this organ, principally, must be ascribed the intense pain in the head and the vomiting, which is often so distressing; the rapid gaseous pulse, and many other most alarming symptoms. Of these, one of the most formidable is the obstinate and profuse secretion of yellow bile, which continues until the whole surface is tinged yellow, and the vital fluids are impoverished beyond the power of sustaining life. This condition becomes daily aggravated, until delirium and death close the scene.

This state of things, in the early stage of the disease, is allayed more by permanently reducing the temperature of the head by cold water, than by any other single means. Opiates, as before observed, are indispensable; and saline laxatives to carry off the morbid secretions. Opiates are too much neglected by many in treating these cases, from a prejudice against its use. This prejudice is founded upon an error in judgment, whereby irritable excitement is confounded with sthenic excitement; and the action of opium itself is not understood.

A case now occurs to my mind which was certainly beyond the agency of all known remedies except cold water affusion. The patient was an athletic young man of about thirty-five years of age, of sanguine temperament, and addicted to occasional intoxication. When in this state, the determination to the brain was uncommon, and was invariably attended with a species of delirium. This patient, towards the last of September, was attacked with *bilious fever;* with great determination of blood to the head, and extraordinary heat in the head. After the general circulation was reduced by bloodletting, purging, &c., the heat of the head continued unabated by every means used for its reduction, including even cold affusion moderately used. Finally the cold affusion was used for fifteen minutes at a time, and repeated every hour for at least twenty hours, when the generation of preternatural heat in the head was subdued, and with it the intractable grade of fever which seemed a consequence. For the first fifteen hours, the head would be as hot again as ever within half an hour after each bathing; and the heat was finally subdued by more perseverance than I have ever seen requisite in any other case.

In this case the bilious purging and vomiting were incessant, the eyes red and watery, and the pain in the head excruciating. Nothing appeared to relieve the inflammatory determination and irritation of the brain but the continued use of the cold affusion; and without it he must have died.

5. *Blisters.*—In most cases of fevers, in this climate, there is, either in the early or advanced stages, more or less irritation or sub-acute inflammation in the mucous membrane of the stomach or bowels. Sometimes this will be co-extensive with the attack; and at other times it may be induced by such remedies as may appear necessary in the treatment of the case. Whenever it exists, and from whatever cause, it serves as an additional source of febrile irritation to the general system; sometimes it proves a very troublesome symptom, and to a certain extent precludes the administration of internal remedies. Accordingly it becomes an object of primary importance to prevent the occurrence of such irritation, as well as to remove it, when it has once occurred. For this purpose it is generally advisable, and sometimes indispensably necessary, after the usual depletion by bloodletting and cathartics, and other remedies adapted to the first stage, to apply a large blister over the region of the stomach or abdomen; especially if the train of febrile symptoms is not greatly arrested within the first twenty-four or thirty-six hours. If the blister be thus applied, as soon as it is found that the febrile action is not subdued by the first remedies, and before any permanent irritation is established in the alimentary canal, it will certainly exert a more happy influence upon the disease than if deferred until the irritation becomes established. The irritation which is thus about to be located in the intestinal canal, will be transferred to the surface and be more transient and less injurious; while at the same time it affords a better opportunity of administering internal remedies than would otherwise present. By this means the violence and duration of attacks of fevers are often limited; and such tonics as may be deemed necessary during convalescence can be more safely administered. But, should blistering be deferred until the irritation is established; until the tongue becomes *clean* and *red*, and even dry and glabrous, the beneficial effects of the blister will be less immediate and less perceptible. For these reasons I advise the early application of a large blister to the epigastrium, so soon as it is perceived that the disease is not disposed to yield to the first common remedies. Thus we anticipate and prevent the occurrence of a symptom which retards recovery and convalescence more than any other.

Sometimes, also, there is some degree of inflammation in the *peritoneal coats* of the abdomen and intestines, from the onset; as well as that which often occurs in the advanced stages of all continued or remittent fevers. The slightest tenderness to pressure over the abdomen should warn us of this condition of the peritoneum. In such cases blisters are good and most commonly used; but the most important benefit will be experienced from scarifying and free cupping. This latter is a remedy of very great advantage in internal inflammations, and is entirely too much neglected by the faculty in their common practice. Were it more used, the benefits obtained from it would more than balance the trouble and time requisite in their use. The effect is incomparably greater than that to be obtained from blisters or leeches.

This sub-acute intestinal inflammation in the *progress* of fevers, can not be too closely observed; for it often approaches so insidiously that it is established before its approach is suspected. Hence frequent examinations are requisite, to ascertain the existence of the slightest soreness or tenderness to pressure upon the epigastrium or abdomen; for the *pain or soreness* is often so obscure at first, that it frequently escapes a hasty and partial examination, and often bears a very small proportion to the actual inflammation, or structural læsions. Dr. Armstrong observes relative to "secondary inflammation," "that the sensibility of the nervous system is mostly somewhat diminished *before* the occurrence of inflammation. This secondary irritable inflammation, if not speedily counteracted, rapidly proceeds and keeps up a constant irritation in the nervous and arterial systems, until the case is beyond the hope of recovery; and often being *mistaken for sthenic excitement,* every remedy administered accelerates the catastrophe; and the practitioner is astonished to find his patient sinking under an excitement, apparently without local origin, which he is unable to subdue."

Whenever febrile irritable excitement continues more than a few days without any apparent equivalent cause, it may be suspected strongly that sub-acute intestinal inflammation is wasting the energies of the system; and the most prompt means must be resorted to immediately. A doughy feeling of the abdomen upon pressure will prove the fact unequivocally, whether much pain be present or not. Counter-irritants, but specially scarrifying and cupping over the whole epigastric and abdominal regions should be used freely and repeatedly, as infinitely superior to blisters; which are apt to increase the general irritation.

Blisters to the head, neck and extremities are too often used where the foregoing symptoms prevail; and where the intestinal irritation removed other symptoms will subside. I have seen blisters thus applied to overcome *irritation* of the brain and general system, which would have been speedily relieved by a laxative followed by an opiate in a little weak ginger toddy. As before remarked, we often find cases of prostrated fever, which by a free use of purgatives or other antiphlogistic medicines, have become cases of debility and irritation; which, if the bowels have not been neglected, will require only rest, anodynes and gentle stimulants of weak brandy toddy. Thus the tone of the circulation is sustained, and the nervous irritation calmed: the reverse would be produced by the antiphlogistic treatment. Calomel especially is pernicious; but opiates in small quantities and of the mildest preparations are indispensably necessary: the prejudice which obtains against them is applicable only to large doses, when administered in improper cases. The most salutary mode of exhibition in most cases is by enema; so that the anodyne effects are obtained without impairing the tone of the stomach. This mode is especially adapted to those cases which are attended with great irritation of the mucous membrane of the lower intestines.

6. *Sulphate of Quinine.*—A few remarks on this powerful febrifuge may not be superfluous. This medicine evidently possesses the properties of subduing febrile action when it does not depend upon local irritations. It is adapted to the treatment of open fever, where the necessary depletion and evacuants have been properly employed. But it is inapplicable in *bilious* fever, or in fevers with gastro-intestinal irritation. In bilious fever the head suffers much from full doses of quinine; and where there is a tendency to intestinal irriattion, the quinine speedily produces dryness in the mouth, with a dry red tongue, and general irritation. It is to be regretted that some physicians, influenced by preconceived notions rather than by observation, are in the habit of giving quinine in doses of from five to ten grains every three or four hours, in every variety of fever, and with but little previous preparation. Doubtless it is much safer to use it thus freely in the early stages while there is no established local irritation, than subsequently when these irritations become located.

The calisaya bark either in substance or in infusion is far less apt to excite intestinal irritation than the quinine; in all cases it is equally efficient in producing a remission of fever, and never excites an irritable state of the arterial system. It possesses all the febrifuge properties of the quinine, together with some valuable aromatic property which corrects any of the irritating effects produced by quinine alone. In combination with the bark I use some aromatic as ginger, pimento, or a small portion of capsicum; and to each dose add from five to ten drops of laudanum. This combination is admirable in *bilious* fever, in which quinine is entirely inadmissible. It is unnecessary to say that the calisaya bark certainly possesses properties very different from those of common cinchona. In cases of bilious remittents, after previous evacuants, I have often given the calisaya bark with a few drops of laudanum and some aromatic, with the best effects even *during the febrile excitement.*

I will close this article by a few remarks upon the appearance and indications of the tongue in febrile affections. In the treatment of these fevers in all their stages, the *tongue is the great index,* and it should be our criterion in the administration of all our remedies. By it we are enabled to judge of the condition of the stomach and bowels; and of the adaptation of our remedies to the case and of the changes necessary to be made in such as we employ. This index is too seldom consulted in prescribing; and what is still more to be regretted, it is too little studied, and its indications too little known when it is consulted.

From the best observation which I have been able to make in these fevers, the following is the result of my experience as to the indications of the tongue.

1. *A pale moist tongue*, covered with mucus, indicates great torpor in the portal circle, or in the cæliaca; a languid circulation and functional torpor in the viscera of the cæliac circle, especially the stomach and liver; sometimes torpor of the brain also exists, with deficient mental energy. In such cases, whether in the beginning or in the advanced stages of all fevers the free exhibition of aromatic stimulants is proper, and necessary so long as the tongue continues moist and pale. The most appropriate stimulants are capsicum, ginger, oil of black-pepper, and calomel: the latter should be combined with either of the aromatics. If the tongue begin to assume red edges, or become red and dry on its surface, whether rough or smoother, the stimulants of every kind should be discontinued; and demulcents and mucilaginous drinks substituted; and as medicines, weak solutions of neutral salts, &c. The incipient redness indicates a change of condition from torpor to one of increased action or increased sensibility. So long as the tongue continues pale and moist, the capsicum and calomel are applicable; but after that ceases neither calomel nor stimulants are proper.

2. *A white tongue*, or a tongue of its natural healthy color, but covered over its surface with a fine white fur, not unlike white powder sprinkled over it, indicates a less degree of visceral torpor, and some degree of irritation in the mucous surface of the stomach. In such cases the stomach will not bear so free exhibition of stimulants as the white moist tongue; and a small quantity of opiate should be combined with any medicine. Calomel in such cases should be combined with opium and ipecac in small quantities; and mild opiates should be combined with all febrifuges.

3. *A red tongue*, moist and raw, indicates extreme irritation in the mucous membrane of the stomach, amounting almost to subacute inflammation of the same, but unattended with congestion of the collatitious viscera. When the other coats of the stomach participate in the inflammation the tongue becomes dry. This variety of tongue will bear only demulcents or mucilages, and other mild vegetable infusions, with mild opiates in very small quantities. As medicines, weak infusions of ipecac, sulphate of magnesia and tinct. opii, all in small quantities, are proper to allay the irritation. Alcoholic stimulants are injurious. Calomel is also injurious, and so is quinine in a high degree.

4. *A dry, red, chapped tongue* indicates that the inflammation is phlegmasial and affects all the coats. The treatment is the same. Scarifying and cupping over the epigastrium is very requisite; or blisters may be used—a free use of demulcents and mild astringent vegetable infusions are also beneficial.

5. *A red, dry, and smooth*, or glabrous tongue indicates a similar state of the coats of the stomach, but more especially of the mucous coat, differing probably only in degree.

The treatment requisite differs nothing from the others.

6. *A brown tongue*, moist and covered with a brown thick fur. This indicates a degree of irritation in the lower bowels while the action in the stomach is rather defective. This appearance of tongue is observed where irritation has been transferred from the stomach and small intestines to the large intestines or lower bowels. In cases of this kind saline purgatives, and such medicines as operate mildly upon the large intestines, are most applicable, together with such tonic vegetable infusions as may serve to rouse the action of the stomach in some small degree. In such cases, other circumstances favourable, quinine would be proper. Resinous purgatives, and such medicines as act harshly upon the large intestines are improper. Scarifying and cupping over the hypogastric region may be necessary.

In the progress of any case of fever the indications of the tongue should be closely observed, and the remedies changed according to the varying symptoms of the disease; especially as it affects the coats of the stomach and bowels.

*Washington, Miss.*, 1839.

